# YKL-40 in the diagnosis, prediction of prognosis, and platinum sensitivity in serous epithelial ovarian cancer

**DOI:** 10.4274/tjod.28459

**Published:** 2018-09-03

**Authors:** İlker Kahramanoğlu, Nedim Tokgözoğlu, Hasan Turan, Veysel Şal, Gönül Şimşek, Remise Gelişgen, Tugan Beşe, Fuat Demirkıran, Macit Arvas, Hafize Uzun

**Affiliations:** 1 İstanbul University Cerrahpaşa Faculty of Medicine, Department of Obstetrics and Gynaecology, Division of Gynecologic Oncology, İstanbul, Turkey; 2 İstanbul University Cerrahpaşa Faculty of Medicine, Department of Physiology, İstanbul, Turkey; 3 İstanbul University Cerrahpaşa Faculty of Medicine, Department of Medical Biochemistry, İstanbul, Turkey

**Keywords:** YKL-40, ovarian cancer, biomarker, platinum sensitivity

## Abstract

**Objective:**

To evaluate the use of YKL-40 in the discrimination between benign and malignant adnexal mass and to determine its prognostic value in assessing residual tumor after primary cytoreduction and platinum sensitivity in serous epithelial ovarian carcinoma (EOC).

**Materials and Methods:**

During the three years from January 2015 to December 2017, a nonconsecutive series of 100 patient (60 malignant, 40 benign) who underwent surgery for an adnexal mass were enrolled in the study. Preoperatively, serum samples were collected for YKL-40 level analysis.

**Results:**

YKL-40 [receiver operator characteristics (ROC)-area under curve (AUC)=0.83] was a significantly better predictor of EOC than cancer antigen-125 (ROC-AUC=0.75). Using a cut-off for YKL-40 of 47.7 ng/mL had a sensitivity of 80% and a specificity of 70%. Higher serum YKL-40 levels were associated with advanced stage, higher grade, residual tumor after primary cytoreduction and recurrence. Platinum-sensitive patients had significantly elevated levels of YKL-40 compared with platinum-resistant or refractory patients.

**Conclusion:**

The results obtained from our study support the use of serum YKL-40 for the discrimination between malignant and benign ovarian tumors. YKL-40 levels in patients with serous EOC may also predict disease residual disease after primary cytoreduction and recurrence. Further studies are needed to understand the relationship between YKL-40 and platinum sensitivity.

**PRECIS:** YKL-40 may be used in the management of adnexal masses and may predict residual disease after primary cytoreduction and recurrence.

## Introduction

Ovarian cancer is a common gynecologic malignancy associated with poor prognosis worldwide. Patients with early stage ovarian cancer have a 5-year overall survival of 70-90%. However, more than 70% of women are diagnosed as having stage III or IV disease, which has a 5-year survival rate of around only 25%^(1)^. This poor prognosis emphasizes the need for early detection in screening programs and prognostic markers to guide individual cancer treatment. The standard treatment of early epithelial ovarian carcinoma (EOC) is full staging including pelvic and paraaortic lymphadenectomy. Debulking surgery followed by platinum-based chemotherapy is the cornerstone of treatment of advanced EOC^(2)^. Neoadjuvant chemotherapy followed by interval cytoreduction has been a debatable alternative option. A metaanalysis of 21 non-randomized trials concluded that survival between two groups was similar^(3)^. Subsequently, Vergote et al.^(4) ^performed a randomized trial including patients with stage IIIC-IV EOC and found that optimal cytoreduction was higher in an interval cytoreduction group compared with a primary cytoreduction group with significantly less morbidity in the interval cytoreduction group. However, a number of studies showed that leaving no residual tumor following primary debulking surgery was the single most important independent prognostic factor in advanced EOC^(4,5)^. Patients who are at higher risk of residual tumor after primary cytoreduction should be isolated in the preoperative period, and these should benefit from neoadjuvant chemotherapy. A glycoprotein, YKL-40, also known as chitinase-3-like 1 protein, was identified in the 1990s. Since then, it has been of special interest because it is associated with degradation of extracellular matrix and promotes angiogenesis in a vascular endothelial growth factor (VEGF)-independent manner^(6,7)^. During the last decade, YKL-40 has been increasingly studied in several tumor types^(6,8,9,10)^. *In vitro*, YKL-40 upregulates VEGF and is associated with tumor angiogenesis^(9)^. *In vivo* animal studies demonstrated that inhibition of YKL-40 decreased angiogenesis, tumor formation, and metastasis^(11,12,13)^. Recent studies showed that neutrophils and tumour cells expressed and released YKL-40 into the blood^(9,14)^. Increased serum levels of YKL-40 were shown in some cancer types such as breast cancer, lung cancer, colorectal cancer, melanoma, and endometrial cancer^(6,7,10,15,16)^. YKL-40 was suggested to have the potential to be a better marker than cancer antigen-125 (CA-125) for early diagnosis of EOC^(17,18)^. However, results from recent studies on the diagnostic efficiency of YKL-40 were inconsistent^(18,19,20)^. There are a limited number of studies reporting that elevated plasma YKL-40 levels are associated with worse outcomes in patients with ovarian cancer^(17,21,22,23)^. Also, it has been suggested that future studies should focus on determining an optimal cut-off value in patients with ovarian cancer for serum YKL-40^(6)^. In this study, we aimed to evaluate the usefulness of YKL-40 in the discrimination of benign and malignant adnexal masses and to determine the efficacy of YKL-40 in the preoperative estimation of the prognostic parameters such as stage and grade of the disease, residual tumor after primary cytoreduction, and response to platinum-based chemotherapy.

## Materials and Methods

This prospective observational study was conducted at İstanbul University Cerrahpaşa Faculty of Medicine, Division of Gynecologic Oncology, between January 2015 and December 2017. The study was approved by the Ethics Committee of İstanbul University Cerrahpaşa Faculty of Medicine (protocol number: 83045809-604.01.02). Written informed consent was obtained from all patients. The manuscript was prepared in accordance with the Strengthening the Reporting of Observational Studies in Epidemiology statement^(24)^. Blood samples were collected preoperatively from a nonconsecutive series of 100 patients who were planned to undergo surgery for an adnexal mass in our clinic. Exclusion criteria were the presence of one or more of the following: i) a suspicous malignancy other than ovarian cancer; ii) systematic disease including renal and/or hepatic impairment; iii) neoadjuvant chemotherapy; iv) history of any malignancy; v) ovarian malignancy other than serous histopathology; and vi) pregnancy. Frozen section evaluation was performed intraoperatively in the presence of suspicion in the diagnosis. The same gynecologic pathologists evaluated all of the specimens. The stage of disease and histologic types were in accordance with the International Federation of Gynecology and Obstetrics classification^(25)^. Maximal cytoreduction was defined as removing all gross tumoral tissue with no visible disease left. Optimal cytoreduction was defined as residual volume of 1 cm or less after surgery. Residual tumor more than 1 cm was classified as suboptimal cytoreduction. All patients with serous EOC (except those with stage IA and IB disease) received 6 cycles of adjuvant carboplatinum and paclitaxel. The platinum-free interval was defined as the interval from the last treatment with platinum to recurrence. Patients were accepted as platinum sensitive, if the platinum-free interval was longer than 6 months; platinum resistant, if it was shorter than 6 months; or platinum refractory, if the disease was persistent or progressive during the first-line chemotherapy. Progression of disease was diagnosed in the presence of elevated CA-125 and imaging results according to Response Evaluation Criteria in Solid Tumors criteria^(26)^. Progression-free survival was defined as the time interval between primary surgery and progression or death from any cause. Blood samples were collected in EDTA-containing tubes and anticoagulant-free tubes after an overnight fast on the morning before the surgery. Plasma and serum were seperated immediately and stored at -80 ºC until analysis. After reaching the desired number of cases in both groups, all serum samples were defrosted at room temperature in the medical biochemistry laboratory of the faculty. Serum YKL-40 concentrations were determined by a commercial enzyme-linked immunosorbent (ELISA) kit using a double-antibody sandwich enzyme immunoassay technique (human chitinase-3-like protein 1 YKL-40, ELISA Kit, Cat. No. YHB0684Hu; Shanghai Yehua Biological Technology Co. Ltd, China). Each ELISA analysis was performed according to the manufacturer’s instructions. All tests showed intraassay and interassay coefficients of variations below 6% (n=10) and 7.5% (n=10), respectively. The analytical sensitivity of the test was 0.52 ng/mL.

### Statistical Analysis

Patients’ characteristics and clinical features were summarized using standard descriptive statistics. Mann-Whitney U test was used for comparison between two groups. T-test was used in comparison of independent samples’ average. Receiver operating characteristics curves (ROC) were constructed for YKL-40 and CA-125 serum concentrations as diagnostics for cancer by plotting sensitivity versus 1-specificity, and area under curve (AUC) was calculated for both markers. All p-values were two sided and p<0.05 were considered as statistically significant. Statistical analyses were performed using SPSS version 21.

## Results

Age, BMI, and menopausal status were similar between the malignant and benign groups (Table 1). The pathologic subtypes of benign ovarian tumors included endometriotic cysts (n=14), serous cystadenoma (n=12), dermoid cyst (n=9), and mucinous cystadenoma (n=5). Serum YKL-40 and CA-125 levels were significantly higher in patients with serous EOC, but CA-19-9 and CA-15-3 levels did not differ between the groups.

**Figure 1:**
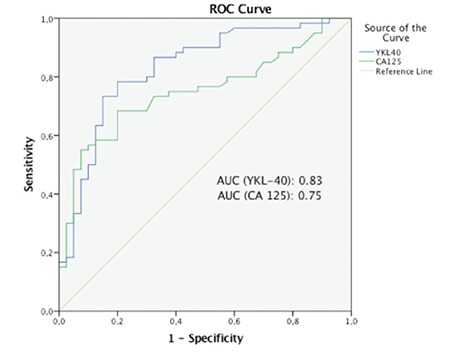
Receiver operating characteristic curves showing the performance of serum YKL-40 and cancer antigen-125 levels for differentiating between benign ovarian tumors and epithelial ovarian carcinoma ROC: Receiver operating characteristic, CA-125: Cancer antigen-125, AUC: Area under curve

**Figure 2:**
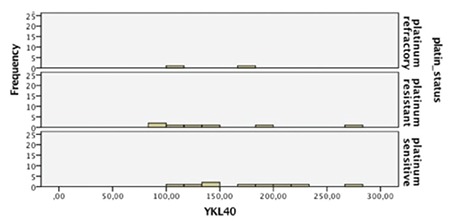
Receiver operating characteristic curves showing the performance of serum YKL-40 and cancer antigen-125 levels for differentiating between benign ovarian tumors and epithelial ovarian carcinoma

As illustrated in Figure 1, sensitivity was 80% and specificity was 70%, respectively, when a cut-off serum YKL-40 level of 47.7 ng/mL was applied. When 34.3 ng/mL was used as a cut-off value, the specificity decreased to 58.5%; however, sensitivity increased to 90%. The obtained areas under the ROC curves for YKL-40 (AUC=0.83) were greater than for CA-125 (AUC=0.75). Patients with serous EOC were analyzed seperately. One-quarter of the patients had stage I disease. Stage III disease consisted of almost 70% of the malignant group. In the next step, we analyzed the relationship between preoperative serum YKL-40 and clinicopathologic features of patients with serous EOC, such as stage, grade, residual tumor, and recurrence (Table 2). Significantly increased serum levels of YKL-40 were observed in patients with advanced stage and higher grade disease. When compared with maximal cytoreduction, optimal and suboptimal cytoreductions were found to be correlated with greater YKL-40 levels. The overall recurrence rate was 30% within a median follow-up time of 21 months (range, 2-35 months). The median time to recurrence after primary surgery and platinum-based chemotherapy was 7 months. The comparison of preoperative serum YKL-40 levels between patients with and without recurrence demonstrated signicificantly elevated levels in the latter patients (Table 2). When patients who had recurrence were investiagted in terms of platinum sensitivity, it was found that YKL-40 were significantly higher in the sensitive group compared with resistant and refractory groups (mean values of YKL-40: 179.2, 146.9 and 141.7 in platinum-sensitve, resistant, and refractory patients, respectively) (Figure 2).

## Discussion

There are still limited data investigating serum markers to differentiate between benign and malignant ovarian tumors^(27)^. For long time, the best documented and the most frequently recommended serum marker has been CA-125 in the management of adnexal masses^(28)^. In this study, preoperative serum levels of YKL-40 were assessed in patients with benign adnexal mass, and in patients with serous EOC. One of the major findings of this study is that patients with EOC had significantly higher serum YKL-40 levels compared with those with benign ovarian tumors. Moderate-to-high sensitivity and specificity (80% and 70%, respectively) was found when a cut-off level of 47.7 ng/mL was used. On the other hand, the sensitivity and specificity of CA-125 (cut-off value: 35 U/mL) were 76% and 57.5%, respecitvely. YKL-40 was a better predictor of ovarian cancer than CA-125 in our study population. Similar findings were observed in previous studies. Plasma YKL-40 levels were found to be elevated in more than 70% of patients with ovarian cancer compared with healthy patients^(17,21,22,23)^. As a result, YKL-40 has been included in the “top 9” biomarkers that are capable of discriminating between malignant and benign ovarian diseases^(29)^. Secondly, we investigated the association between YKL-40 and prognostic factors of EOC such as stage and grade of disease, residual tumor after primary cytoreduction, and recurrence. Results from similar studies suggested that high preoperative YKL-40 was an independent predictor of worse survival, which is in accordance with our findings^(6,17,21,22,23)^. Zou et al.^(19)^ found that YKL-40 was associated with poor clinical outcome and worse tumor stages and grades. A unique study from Copenhagen, investigating the prognostic value of plasma YKL-40 in platinum-resistant ovarian cancer patients treated with bevacizumab found that elevated YKL-40 levels (>95^th^ percentile) at baseline were associated with poor survial and residual disease after primary surgery^(6)^. Furthermore, two studies found that high serum levels of YKL-40 were associated with chemoresistance^(22,30)^. On the contrary, higher YKL-40 levels were detected in platinum-sensitive patients in the present study. One possible explanation for the contrast may be that Gronlund et al.^(22)^ examined the role of YKL-40 on chemosensitivity in the second-line treatment of EOC patients, whereas we included only patients with serous EOC who received first-line platinum-based chemotherapy. For the first time, Chudecka-Glaz et al.^(30)^ demonstrated that higher serum YKL-40 levels were associated with first-line platinum resistance. Their study and ours included a limited number of patients. Larger studies are needed to see if there is a correlation between YKL-40 and platinum sensitivity.

### Study Limitations

It has been suggested that future studies of serum YKL-40 should be powered to investigate its value as a biomarker in individual histologic subtypes of ovarian cancer^(6)^. In our study, all patients with ovarian malignancy had the same histopathology. The present study included patients over a period of almost three years. However, because of the short study period, the same practices (same surgical team, same chemotherapy regimen starting on almost same postoperative days) were applied to all patients. The limitations of this study include the small number of patients, which means that strong conclusions cannot be drawn.

## Conclusion

In conclusion, our findings suggest that preoperative serum YKL-40 can be used for to discriminate between malignant and benign ovarian tumors. In addition, higher YKL-40 levels indicate a higher risk of recurrence. Conflicting results regarding YKL-40 and platinum sensitivity should be eliminated by larger prospective studies.

## Figures and Tables

**Table 1:**
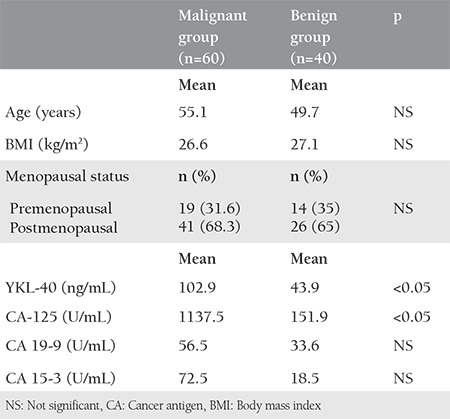
Clinical and laboratory characteristics of groups with malignant and benign disease

**Table 2:**
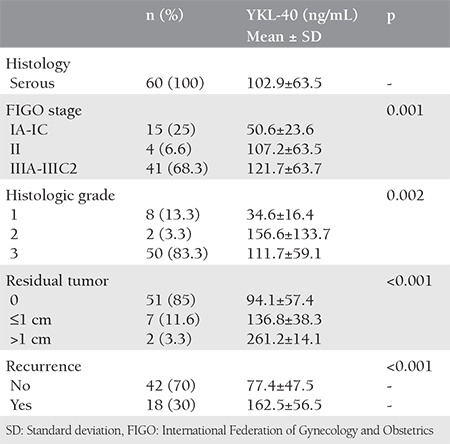
Clinical characteristics and plasma YKL-40 levels of patients with serous epithelial ovarian carcinoma
